# Breast dose heterogeneity in CT-based radiotherapy treatment planning

**DOI:** 10.4103/0971-6203.41191

**Published:** 2008

**Authors:** R. Prabhakar, G. K. Rath, P. K. Julka, T. Ganesh, R. C. Joshi, N. Manoharan

**Affiliations:** Department of Radiotherapy, Institute Rotary Cancer Hospital, All India Institute of Medical Sciences, New Delhi, India

**Keywords:** CT-based planning, dose heterogeneity, radiotherapy

## Abstract

The aim of this study was to evaluate the breast dose heterogeneity in CT-based radiotherapy treatment planning and to correlate with breast parameters. Also, the number of slices required for treatment planning in breast cancer by tangential field technique has been assessed by comparing the treatment plans according to International Commission on Radiation Units and Measurement (ICRU) 50 guidelines (1993) for single-slice, three-slice, and multi-slice (3D) planning . Sixty women who underwent isocentric tangential field breast radiotherapy were included in this study. The plans were optimized and analyzed with dose volume histograms. Sixty-three percent of the single-slice plans and 26.7% of the three-slice plans showed poor dose homogeneity as compared to the 3D plans. Dose inhomogeneity correlated better with breast volume (r^2^ = 0.43) than the chest wall separation (r^2^ = 0.37) and breast area product (r^2^ = 0.36). Similarly, breast volume correlated better with breast area product (r^2^ = 0.80) than with chest wall separation (r^2^ = 0.56). Breast volume can be approximated to breast area product from the relation, breast volume = [(breast area product × 8.85) − 120.05]. The results of this study showed that most of the cases require 3D planning for breast cancer. It also showed that patients with large breast are prone to have more dose inhomogeneity with standard tangential field radiotherapy. In centers where 3D planning is not possible due to lack of facilities or workload, three slices–based planning can be performed to approximate the dosimetric advantage of 3D planning.

## Introduction

Cancer of breast is one of the most common malignancies in women and is the single most common cause of death among women aged between 40 and 50 years. Every year there are about 1 million new cases in the world (20% of total cancer-related diseases), and this comprises 18% of all cancers in women.[1] In India, it is the leading site of cancer among females in all cancer registries except the registry of Barshi, with the relative proportion ranging from 19.3% to 27.5%; and it has overtaken the incidence of cervical cancer in recent years.[2] With advances in mammography and public awareness, breast cancer is being detected at an earlier stage. The combination of lumpectomy and radiotherapy is the standard treatment option for most women with stage I and stage II invasive breast cancer.

The introduction of CT scanning and the availability of sophisticated three-dimensional treatment planning methods have improved the delivery of radiation to the breast efficiently. A number of important and challenging technical issues have been shown to influence the successful outcome of this therapy. These include i) dose distribution and homogeneity, ii) coverage of highly complex target and setup.[3,4] Radiotherapy treatment fields are usually tangential to encompass the breast, and, in some cases, matched to a supraclavicular field. Hurkmans *et al.*, in their study on intra- and inter-observer variability of the target volume of breast tumor on CT scan, showed that intra-and inter-observer variability was reduced with lead wires placed around the palpable breast.[5] Due to variation of shape and size of the breast, dosimetry and treatment planning can be challenging. Many radiotherapy centers still achieve a treatment plan for the breast by using a single-plane hand-generated contour through the center of the breast (2D planning). This type of planning neglects the variations in contours and chest-wall separation in the other plane, which has significant impact on dose homogeneity. With the availability of CT-scanner, most of the centers are gradually shifting towards CT-based treatment planning; and more often, planning is performed on a single slice or on three slices. CT-based three-dimensional treatment planning allows the planner and physician to evaluate the dosimetry across the entire breast. Consequently the plan can be optimized to limit lung volume with selective blocking and minimize hot spots by using a higher energy and lesser wedge angle. According to ICRU-50 guidelines, an optimal plan is one in which the entire planning target volume (PTV) is between 95% and 107% levels relative to 100% prescription point.[6] The main difficulty with tangential field breast irradiation is in the achievement of homogeneous dose distribution inside the target volume. Dose heterogeneity is believed to be one of the main contributing factors responsible for poor cosmesis and complications,[7] besides higher skin dose and scatter contribution from the collimator. It has been demonstrated that cosmetic effect is more likely to be poor, and the frequency is higher for lung fibrosis in large patients[8] This may be possibly due to greater dose inhomogeneity[9] Intact breast treated by conventional tangential fields, requires medial and lateral wedges to improve the dose inhomogeneity because of its pyramidal shape. There is some concern that the radiation scatter from the medial wedge may contribute to cancer induction in the contralateral breast.

In 3D planning, the two slices away from the central plane — 1 cm above the lower field edge and 1 cm below the upper field edge — are the regions where high dose volumes are most likely to occur, and this gives rise to the bulk of dose inhomogeneity.[10] One of the important issues in most of the centers is the number of slices required for treatment planning in breast cancer. In routine radiation therapy treatment planning for breast cancer, a single slice is taken at the center of the tangential field marked on the skin surface of the patient. Some centers use three slices, one along the central plane and two slices between 1 cm and 1.5 cm on inferior and superior field borders. In 3D planning, contiguous slices are taken with a margin from the lower field border to the upper field border.

This study has been focused on the patient-specific factors affecting the dose homogeneity inside the target volume. The aims of this study were twofold: a) to correlate the breast dose heterogeneity with different breast parameters such as chest wall separation, breast area product, and breast volume; b) to study the number of CT slices required for treatment planning in tangential field breast radiotherapy and to establish a simple relation for estimating the breast volume; and accordingly. one can decide upon the cases that require mandatory 3D planning.

## Materials and Methods

In this study, the number of slices required for planning breast cancer by tangential field technique has been assessed by comparing the treatment plans according to ICRU-50 guidelines (1993) for single-slice, three-slice, and multi-slice planning. The comparison was performed based on the target volume receiving >107% and <95% of the dose normalized to the reference point (reference point being the isocenter). Sixty patients treated from October 2004 to June 2005 in our hospital were selected for this study. Somatom™ Volume Zoom CT-scanner (Siemens Medical Systems) was used for imaging the patients. CT scanning was performed for all the patients with 2.5-mm slice thicknesses. Reference markers were placed on the medial and lateral borders of the medial and lateral tangential fields respectively, which aid in contouring the planning target volume. The arm on the treatment side was abducted to 90° to 110°. For 3D treatment planning, an addition of 3-cm margin was taken on both sides of the superior (cranial) and inferior (caudal) border of the field marked by the radiation oncologist. For three-slice planning, three CT slices were taken: (i) along the central slice, (ii) 1 cm to 1.5 cm below the superior field border, and (iii) 1 cm to 1.5 cm above the inferior border. For single-slice planning, a single-CT cut was taken along the center of the marked field. The CT datasets were transferred to the Plato-Sunrise™ (Nucletron B.V.) treatment planning system through DICOM network, and three separate studies were created. A margin of 5 mm was given between the breast planning target volume and the skin surface.[6,11]

All the patients were planned for 6 MV x-rays on a Clinac 2300 C/D™ linear accelerator. Inhomogeneity correction was applied by the equivalent tissue air ratio method for dose calculations. A typical medial and lateral tangential field technique was used for planning, and gantry angles were chosen to achieve a nondivergent posterior beam edge. The treatment plans were optimized using dose volume histogram (DVH), and the plan was normalized to the isocenter. Separate tangential field plans were made for single-slice planning, three-slice planning, and complete 3D-based planning.

**Figure 1:**
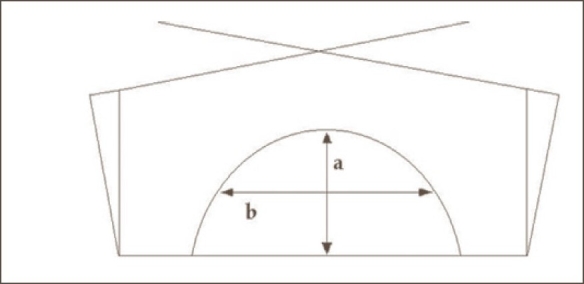
Breast area product calculation method. Line ‘a’ is perpendicularly bisecting the line between medial and lateral field entrance points. Line ‘b’ is perpendicular to ‘a’ and bisects it. Breast area product is the product of ‘a’ and ‘b’

The single-slice and three-slice plans were exported to the complete 3D CT image datasets, and the dose was recalculated without any change in the beam parameters. Dose volume histograms were generated for all the treatment plans. The PTV receiving <95% and >107% were noted for all three plans (single-slice planning superimposed on the 3D CT dataset, three-slice planning superimposed on the 3D CT dataset, and original 3D optimized plans) from DVH and then compared as per ICRU-50 guidelines. Breast volume of the contoured ipsilateral breast was recorded for each patient. Breast area product was calculated for all the patients from the product of two parameters (a and b) measured from a single transverse contour of the breast taken through the isocenter at the central plane of the breast as described by Neal *et al*.[11] Figure 1 shows the method for calculation of the breast area product. The line ‘a’ represents the anterior thickness of the breast as measured by the perpendicular line that bisects the breast into two equal lengths at the projection of the posterior beam edges, and line ‘b’ is the field separation at the central axis of the beams. Breast area product = a ×b. Dose inhomogeneity was correlated with breast volume, breast area product, and chest wall separation. Also, the breast volume was correlated with breast area product and chest wall separation

## Results

**Figure 2:**
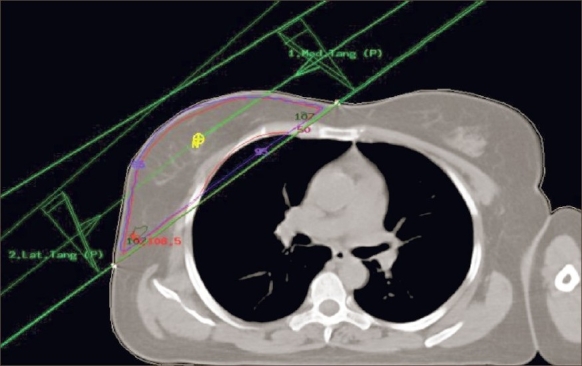
CT-based planning for a target volume of 629 cc

**Figure 3:**
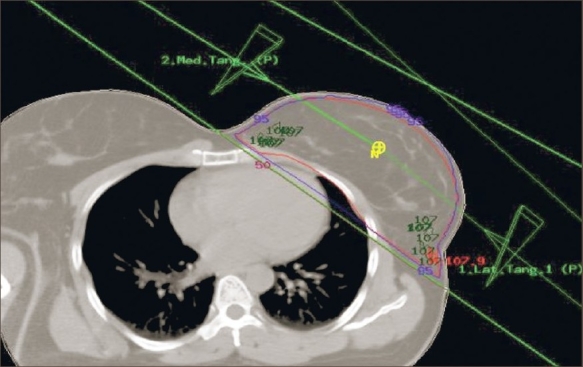
CT-based planning for a target volume of 1,231 cc

**Figure 4:**
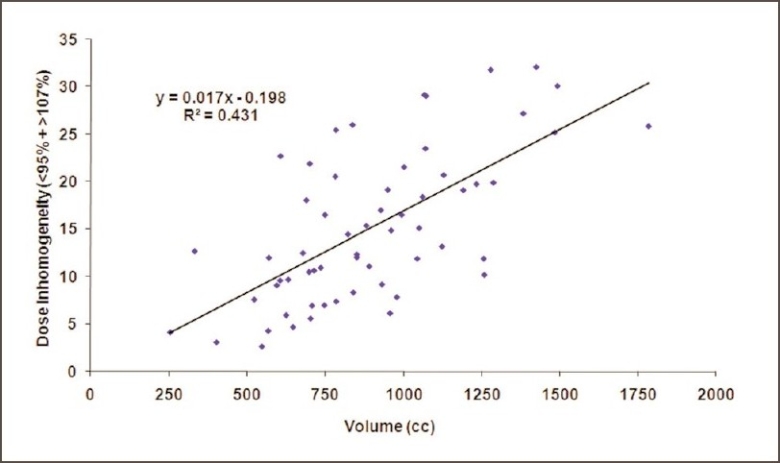
Regression plot of dose heterogeneity (breast volume <95% and >107%) within the target volume against breast volume

**Figure 5:**
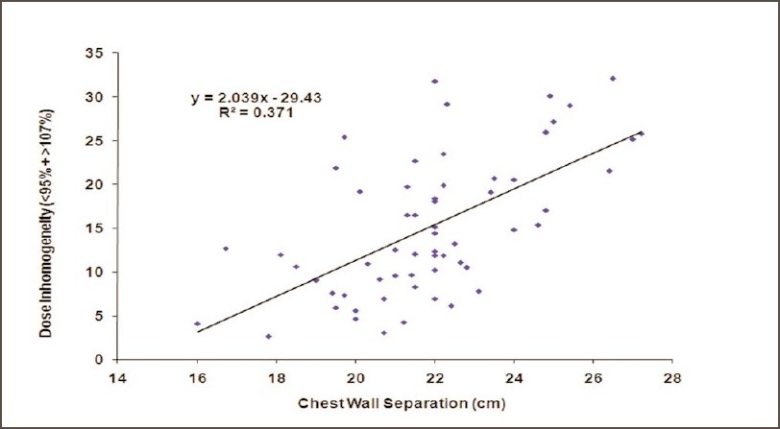
Regression plot of dose heterogeneity (breast volume <95% and >107%) within the target volume against chest wall separation

**Figure 6:**
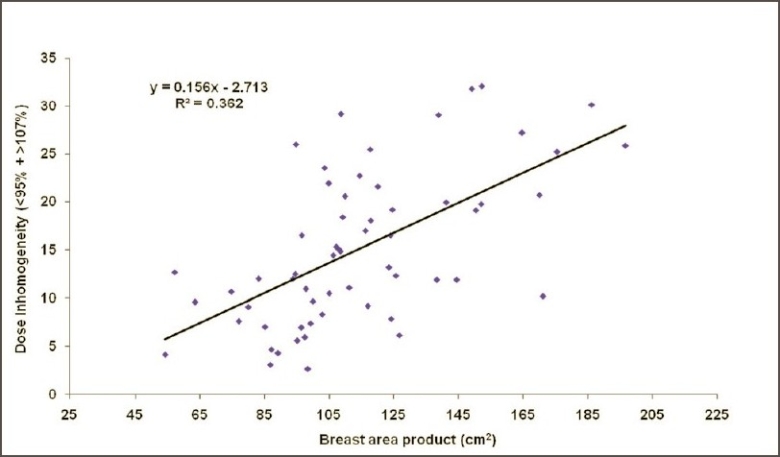
Regression plot of dose heterogeneity (breast volume <95% and >107%) within the target volume against breast area product

**Figure 7:**
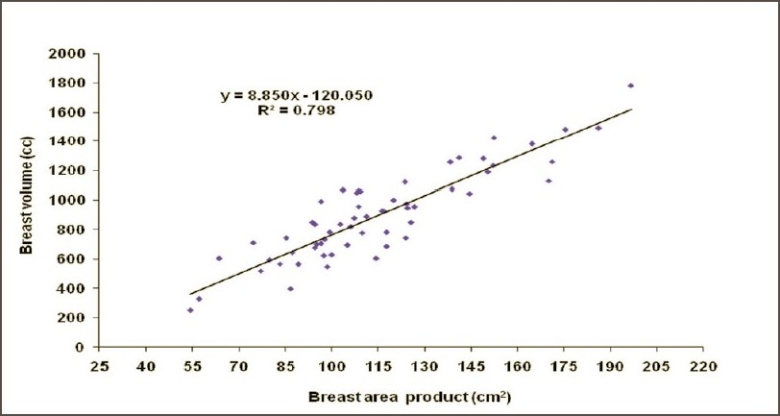
Regression plot of breast volume against breast area product

**Figure 8:**
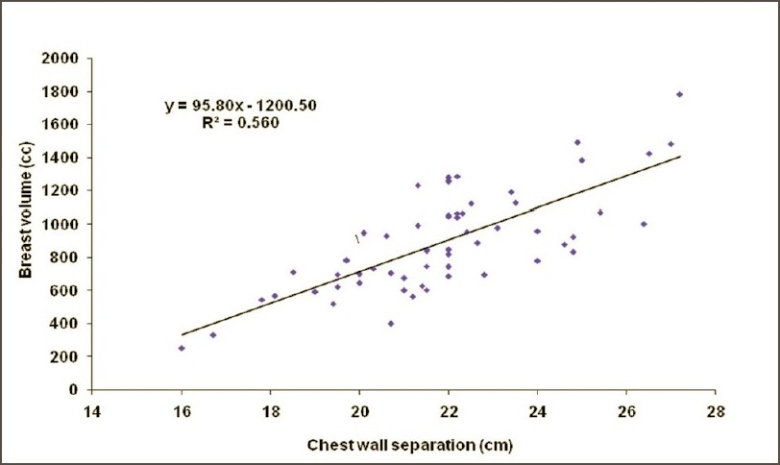
Regression plot of breast volume against chest wall separation

Figures 2 and 3 show the CT-based planning employing tangential field arrangement with matching posterior field borders performed on PTV of 629 cc and 1,231 cc respectively. The total dose inhomogeneity (<95% + >107%) for Figures 2 and 3 were 9% and 20% respectively, indicating the increase in dose inhomogeneity with increasing breast volume. Table 1 shows the dosimetric parameters analyzed in this study. Table 2 shows the relative comparison of single-slice, three-slice plans with the 3D plan in terms of dose homogeneity. It shows that 21.7% of the single-slice plans and 56.7% of the three-slice plans were same as that of the 3D plans (plan performed on completed 3D CT dataset), defined as being within 1% level. Fifteen percent of the single-slice plans and 16.7% of the three-slice plans had better homogeneity as compared to the 3D plans. On the contrary, 63.3% of the single-slice plan and 26.7% of the three-slice plans showed poor dose homogeneity as compared to the 3D plans. These patients would have achieved a better dose homogeneity if they would have been planned using a full set of CT slices. Table 3 shows the distribution of dose inhomogeneity statistics of 3D planning for different ranges of target volume. The volume was compared in intervals of 300 cc with dose inhomogeneity, and the maximum number of patients studied lay in between 651 cc and 950 cc. The total dose inhomogeneity for the treated breast (<95% + >107%) was correlated with the breast volume [Figure 4], chest wall separation [Figure 5], and breast area product [Figure 6]. Dose inhomogeneity and breast volume showed a better correlation (r^2^ = 0.43), followed by chest wall separation (r^2^ = 0.37) and breast area product (r^2^ = 0.36). This shows that breast volume is a better indicator of dose inhomogeneity as compared to the other two parameters. It also shows that full 3D CT volume is essential for treatment planning. To study the interrelation between the above parameters, breast volume was correlated with chest wall separation and breast area product. Breast volume showed a very strong correlation with breast area product (r^2^ = 0.80) [Figure 7] as compared to chest wall separation (r^2^ = 0.56) [Figure 8]. This shows that breast area product is a better indicator for breast volume in comparison to chest wall separation.

**Table 1:** Breast dosimetric parameters

*Dosimetric parameters*	*Range*	*Median*	*Mean ± Stdev*
Chest wall separation (cm)	16.0 - 27.2	22.0	21.9 ± 2.4
Breast area product (cm^2^)	54.2 - 196.6	108.65	114.5 ± 30.6
Volume (cc)	251.0 - 1782.6	848.7	893.4 ± 302.6
Dose inhomogeneity for single slice plans (<95% + >107%)	3.0 - 37.4	19.0	19.5 ± 7.9
Dose inhomogeneity for 3-slice plans (<95% + >107%)	3.0 - 33.4	15.7	16.7 ± 7.5
Dose inhomogeneity for 3D plans (<95% + >107%)	2.6 - 32.1	12.9	15.0 ± 7.8

**Table 2:** Relative comparison of single-slice, three-slice plans with 3D planning in terms of dose homogeneity

	Single slice plans (%)	Three-slice plans (%)
Same as 3D plans	21.7	56.7
Better than 3D plans	15.0	16.7
Worse than 3D plans	63.3	26.7

**Table 3:** Distribution of dose inhomogeneity statistics of 3D planning for different ranges of target volume

*Volume (cc)*	*No. of patients*	*Total dose inhomogeneity (<95% + >107%)*		
		
		Range (%)	Median (%)	Mean ± stdev (%)
251 - 650	13	2.6 - 22.7	7.6	8.3 ± 5.4
651 - 950	23	5.6 - 26.0	12.4	13.9 ± 5.9
951 - 1250	15	6.2 - 29.2	18.4	17.8 ± 6.7
1251 – 1550	8	10.2 - 32.1	26.2	23.6 ± 8.7
1551 – 1850	1	25.9	25.9	25.9 ± 0.0

## Discussion

Breast is one of the difficult sites in treatment planning, where the dose inhomogeneity could be higher because of the shape and size of the breast, beam energy, and incorporation of inhomogeneity correction. It is widely agreed that inhomogeneity correction should be applied in the treatment planning of breast cancer.[9,12] Our experience shows that breast planning on three slices is better than single-slice planning. The three-slice planning almost approximates to breast planning done with a complete 3D breast volume. It is better to analyze the dose distribution slice by slice, which gives a clear picture of the high dose region. At the same time, one can replan if the underdosage or hot spots are not within the acceptable limits. This study reveals that single-slice planning is unsuitable in most of the cases, and planning on three slices can be performed for most of the cases except for large breast, where 3D treatment planning is mandatory. The results clearly indicate that a 3D approach is better than selecting single slice or three slices for treatment planning in tangential field radiotherapy. The comparison of three-slice plan and 3D planning showed that 56.7% of the plans were within ±1% level, and 16.7% of the three-slice plans showed better dose homogeneity than 3D plans. This indicates that 73% of the patients can benefit from three-slice plan; hence in situations where a complete set of CT slices are not available, at least a minimum of three slices should be used for planning.

Moody *et al.* observed a significant correlation between the breast size and dose inhomogeneity and concluded that it may account for the marked changes in breast appearance reported in women with large breasts.[7] Neal *et al.* observed similar results, that large-breasted women are more likely to have more heterogeneous dose distributions, and stated that breast remnant volume of <600 cc and/or A or B bra cup size is associated with a low probability of a very inhomogeneous dose distribution.[10] Neal *et al.* stated that breast dose heterogeneity most strongly correlated with breast volume (r = 0.7), and there was a positive correlation of breast dose heterogeneity with bra cup size, breast size (r = 0.39), and chest wall separation (r = 0.31). They concluded that breast size is an important parameter for dose heterogeneity within the breast.[13] We found similar results for breast volume, but the dose inhomogeneity also correlated better with chest wall separation. It shows that dose inhomogeneity is directly related to the chest wall separation, as shown by Das *et al*.[14,15]

Cheng *et al*. in their study on the number of CT slices on dose distribution concluded that for patients whose breast contours vary slowly within the tangential fields, a three-slice CT scan, as well as a pseudo-3D approach, would be adequate.[16] For patients with large variation of contours within the tangential fields, a full-scale CT scan with a true 3D dose algorithm is more accurate than either the three-slice or the five-slice model. Vincent *et al*. in their study on 41 patients showed that single-slice plan is unsatisfactory in providing sufficient information about the dose variation across treatment volume and that ideally a 3D plan with DVHs should be produced. They concluded that at least a minimum of three CT slices should be used as an approximation.[17] Our study also showed similar results.

Dose heterogeneity may have several consequences. Low dose volumes within the target volume of the breast result in reduced tumor control probability, and the magnitude of this effect depends on the amount of macroscopic and microscopic residual disease; whereas high dose volumes could lead to increased late normal tissue morbidity. Retrospective data show that the reduction in breast dose from 50 Gy to 45 Gy may lead to reduction in local control from 95% to 85%.[18] Das *et al*. have clearly illustrated that chest wall separation was the most important parameter which correlated with the hot spot and have also shown that for patients with large chest wall separation, energy higher than 6 MV will reduce the hot spot.[14,15] Patients with large breasts are shown to have a worse cosmetic result, and this is believed to be a consequence of greater dose heterogeneity[7,19,20] Dose inhomogeneity with increasing breast volume indicates that 3D planning is mandatory for large breasts. In those cases where inhomogeneity is a serious concern, especially for large breasts, planning with field-in-field technique or with intensity-modulated radiation therapy (IMRT) may reduce the high-dose regions inside the target volume. From our study, it can be observed that for volume >951 cc, the dose inhomogeneity exceeds by more than 15%, indicating that it may require either forward or inverse IMRT.

Our study shows that breast volume may be obtained by correlating the breast area product with the breast volume as shown by Figure 7 (r^2^ = 0.80; p < 0.0001):

Eq. (1) Breast volume=(breast area product × 8.55)-120.05

This study also shows that breast volume is an important parameter for indicating the dose heterogeneity inside the target volume. Even in radiotherapy centers where single slice– or three slices–based planning is performed, breast volume may be roughly calculated from Eq. (1). This may help the radiation oncologist to identify those cases that require compulsory 3D planning.

## Conclusion

A dosimetric comparison of tangential breast planning showed that most of the cases require 3D planning for breast cancer. In centers where 3D planning is not available or if there is a heavy workload, three slices–based planning can be done to approximate the dosimetric advantage of 3D planning. The study also shows that it is difficult to achieve dose homogeneity as prescribed by ICRU-50 report, even with 3D planning, and an optimal plan close to ICRU guidelines is possible for tangential field breast technique. The positive correlation between dose heterogeneity and breast volume showed that women with large breasts are likely to have high dose heterogeneity. Treatment plans options for large-breasted women should be carefully analyzed and planned for acceptable dose heterogeneity.
